# The Effects of Public Open Space on Older People’s Well-Being: From Neighborhood Social Cohesion to Place Dependence

**DOI:** 10.3390/ijerph192316170

**Published:** 2022-12-02

**Authors:** Shi Chen, Yi Sun, Bo Kyong Seo

**Affiliations:** 1 Department of Building and Real Estate, The Hong Kong Polytechnic University, Hong Kong, China; 2 Department of Applied Social Sciences, Centre for Social Policy and Social Entrepreneurship, The Hong Kong Polytechnic University, Hong Kong, China

**Keywords:** public open space (POS), neighborhood social cohesion (NSC), place dependence (PD), place attachment, well-being

## Abstract

This quantitative study examines the effects of Public Open Space (POS) on older people’s well-being and examines the roles of neighborhood social cohesion (NSC) and place dependence (PD) as series buffers. A questionnaire survey of 501 people aged 65 and over was conducted in various communities of Hong Kong. Structural equation modelling (SEM) was used to analyze the pathways connecting POS and well-being. A multigroup analysis examined differences in the POS–well-being associations between the young-old (aged 65 to 75, *n* = 166) and old-old group (aged 76 to 95, *n* = 166). Results show that the association between POS and emotional well-being was stronger than social and psychological well-being. POS promotes three facets of well-being through developing NSC and, subsequently, PD. Multigroup analysis results suggest that the pathway from POS to emotional well-being via NSC is stronger for the old-old group; POS is more important for psychological well-being for the young-old group. This study highlights that the quality of POS, including attractive natural elements, various amenities, and sufficient space for social interactions, is essential for making relationship-rich and health-promotive urban environments.

## 1. Introduction

**Figure 1:**
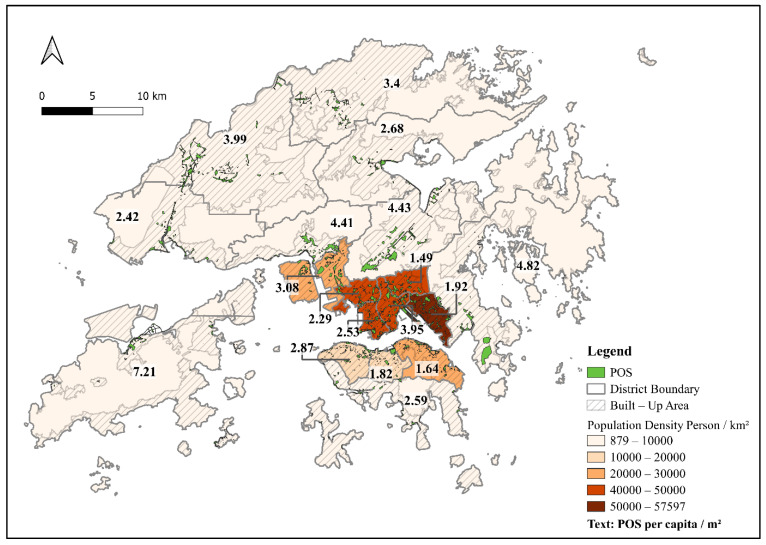
Public open space distribution and coverage (by per capita) in Hong Kong.

Hong Kong is one of the world’s densest cities, with approximately 7.5 million residents accommodated in only 7% of the territory (about 1114 km^2^) [1]. The insufficient land and compact urban developments have led to exceedingly limited availability of, and access to, public open space (POS) in Hong Kong. POS refers to physical spaces with openness and free access. Specifically, POS includes streets, squares, parks, gardens, and other forms of urban green spaces [2]. On average, there is only 2.8 m^2^ of POS per person in Hong Kong, which is far less than in other densely developed Asian cities, such as Tokyo (5.8 m^2^) and Singapore (7.4 m^2^) [3]. In addition, there is an uneven distribution of POS in the city: in the districts with the highest population density (e.g., Wong Tai Sin, Kwun Tong in Figure 1), POS per person is the lowest, at less than the 2 m^2^ standard of the Hong Kong Planning Department [4]. Around 24.6% of Hong Kong’s urban population (about 1.84 million) have less than 2 m^2^ of POS per person in their neighborhoods [3]. Therefore, maximizing the benefits of the limited POS in the high-density built environment is an important target for urban planning in Hong Kong [5].

Researchers have found that POS provides a venue for restoration and recreation, and serves as a gathering place for people to enjoy leisure activities, recreation, and socializing [6,7]. In addition, a good-quality POS can promote the connection to nature, improve residents’ quality of life and residential satisfaction, and enhance the urban landscape [8,9]. The literature highlights that POS is particularly beneficial to older people, who are susceptible to the loss of friends and partners, social isolation, and loneliness when aging in place [10,11]. POS facilitates physical activities and social interactions, conducive to people’s well-being [12]. 

While extensive literature has established the relationship between POS and older people’s well-being, little is known regarding the underlying mechanisms. According to Smith (2009), the socio-spatial features of the environments are “cues” for older people to develop various psychological experiences, which influence their understandings of and attitudes toward where they live [13]. For example, a comfortable and inviting POS will attract people to use it, thereby creating chances for social interactions and developing social cohesion [14]. Positive psychological experiences further trigger older people’s behavioral intentions (such as physical activities and social interactions) and positive emotions, promoting their well-being. 

For the psychological experience relevant to older people’s interaction with POS, this study pays attention to neighborhood social cohesion (NSC) and place attachment. NSC, denoting positive relationships among community members [15], is crucial for older people since it helps counteract their social isolation after retirement or the loss of friends/partners [16]. Place attachment, typically referring to people-place bonding whereby people develop special feelings and emotions towards a particular geographic location [17,18], can be formed when older people feel connected to and dependent on where they live [19,20]. However, for a long time, planners and policymakers seem to pay more attention to the physical features of POS. Understandings of the possible psychological benefits are insufficient in the planning, design, and management of POS [21]. For example, a study shows that POS in Hong Kong lacks amenities. It is difficult to find and is uninviting [22]. In fact, all these drawbacks may hinder the development of positive psychological experiences which further influence older people’s well-being. 

It appears that neither NSC nor place attachment affects older people’s experience of aging independently. For example, a study conducted in the US found that place attachment is positively related to a higher sense of NSC [23]. A British study suggested that the sense of familiarity and social connections with the place of residence contributes to place attachment [24]. However, existing studies seem to pay insufficient attention to mechanisms underlying the linkages between people’s environmental encounters and well-being, especially mechanisms at people’s different life stages [22,25,26,27].

In this respect, this study seeks to develop a comprehensive understanding of the pathways linking POS and the well-being of older people. The following research questions are addressed: (1) How is POS linked with older people’s well-being, and can NSC and place attachment be explained as two interactive buffers underlying such relationships? (2) Whether the established pathways from POS to older people’s well-being differ for different age groups (i.e., young-old vs. old-old)? 

To answer these research questions, structural equation modeling (SEM) and multigroup analysis were used to analyze data from a questionnaire survey of 501 community-dwelling older people aged 65 and above in Hong Kong, one of the most rapidly aging societies around the world (19.6% of Hong Kong’s population is aged 65 or over by 2021, and the number is predicted to increase to 33.3% by 2039 [28,29]). Building an age-friendly environment has been promoted by the government, and enhancing the city’s POS is one of the most important planning initiatives [28]. In line with this policy direction, the study will provide evidence-based suggestions to improve the quality of POS, as well as older people’s positive psychological experiences and well-being.

## 2. Literature Review: From POS to Multifaceted Well-Being

### 2.1. The Direct Impact of POS on Older People’s Well-Being

Achieving well-being is crucial for older people. Well-being means participating actively in society by exploring older people’s potential to live a well-rounded life with autonomy, dignity, and full meaning of life [29,30,31,32]. The meanings of well-being have evolved from its original emphasis on happiness to incorporate multiple aspects, including emotional, social, and psychological well-being [33]. According to Keyes (2007), emotional well-being refers to positive feelings such as happiness and satisfaction [34]. Social well-being relates to the social functioning of people, such as social acceptance and integration [35,36]. Psychological well-being is a complex construct that concerns optimal psychological functioning and experience, such as environmental mastery, autonomy, sense of fulfillment, and emotion regulation [37,38]. 

With particular reference to older people, the presence and use of POS promote their well-being in multiple ways. For example, POS reduces the negative impacts of unpleasant urban environments, such as noise, extreme weather/temperature, and air pollution through enhanced green coverage and dispersion effect [39,40,41]. POS mitigates the loss of nature in urban areas, provides aesthetic and emotional comfort, and allows people to recover from stress and mental illness [42,43]. Older people are likely to develop positive emotions when they live close to POS with sufficient blue and green features [44]. A study in Beijing, a high-density Chinese city, also suggests the associations between a lack of POS and the development of depression symptoms [45]. Viewing and staying close to POS may increase sensory comfort for older people, and thus be conducive to their emotional well-being [46]. 

Active use of POS includes doing more physical activities and engaging actively in social mingling. POS encourages older people to walk out and about [12]. A study in Hong Kong indicates that 75% of recreational walking of older people takes place in POS within the neighborhood, with 42% occurring in parks and 32% occurring on the street [47,48]. Living near urban parks is associated with less likelihood of sedentary behaviors [49]. Older people perceive POS as places for play and recreation, such as stretching and exercise [50,51]. These features increase older people’s sense of emotional well-being and life satisfaction [52]. 

POS provides a space of encounter where people meet and interact with their friends and neighbors [12]. For example, recreation activities like dancing, exercising, and walking pets enhance older people’s social interaction [53,54]. As such, older people get to know more about their neighbors and establish strong bonding ties with where they live [19,20] The social ties with neighbors contribute to social inclusion (referring to the full and fair access to community-based resources and activities, having good relationships with family, friends and acquaintances, and having a sense of belonging to a group, see: [55]), conducive to social well-being [53,56]. 

Visiting POS as a daily occurrence is a reflection of autonomy, as older people have the right to make decisions and govern themselves without being influenced by others [57]. The use of amenities and services such as walkways, benches, and the first aid center in POS can positively affect the self-esteem of older people, as their needs are valued and satisfied, and accepted by their surroundings [58]. Among older people with dementia, nature connectedness is positively associated with risk-taking, personhood support, and autonomy [59]. POS enables older people to feel good about themselves and gain a sense of environmental mastery, leading to better psychological well-being [60].

A growing body of literature acknowledges that experience in aging in place varies at different stages of later life [25,26,27]. The pathways linking POS and well-being are likely to differ between young-old and old-old groups. For example, studies show the difference in terms of physical, cognitive, and socio-emotional functioning (i.e., lower expectations and higher satisfaction that occur with aging, see: [61]) for young-old and old-old groups The young-old group is defined as individuals aged 65–75 who are relatively healthy and independent, while the old-old group is defined as individuals aged 76 or older [62,63,64]. Although aging is associated with significant losses and decline, people’s life experience has become more positive due to better emotion regulation as they age [65]. Studies also show that the old-old group tends to rely more on their immediate environment (like POS) to acquire a good quality of life, as the geographic range of their daily routines is narrower compared with other age groups [66,67]. 

### 2.2. The Relationship between POS and Older People’s Well-Being: The Role of NSC and Place Attachment

Studies uncover that the development of positive psychological experiences, such as NSC and place attachment, is essential for POS to become health-promotive. NSC produces trust, mutual obligations, and respect among neighbors [68,69]. Older people are more likely to feel a sense of belonging and social acceptance if they perceive their neighborhood to be socially cohesive [6,70]. These feelings are essential components of emotional, social, and psychological well-being [71,72]. Various activities and social interactions that happen in POS cultivate NSC [6]. A study in four European countries found that more time spent in POS is associated with social cohesion, which further promotes emotional well-being [73]. Particularly for older people with low socioeconomic status (SES), NSC enhances their well-being by providing chances to receive social support [74]. NSC fulfills their psychological needs of feeling connected and develops a sense of belonging, conducive to their social well-being [6]. 

Place attachment is another possible mechanism underlying the POS–well-being association [17,21,75,76]. While it is widely agreed that place attachment is a multidimensional concept, the literature suggests that place attachment can be analyzed on two key dimensions, namely ‘emotional-symbolic’ meanings and ‘functional’ meanings [75]. The former is termed place identity, and the latter is defined as place dependence (PD) [77,78]. Place identity is an element of self-identification that occurs when people identify themselves through their connection to a particular place [79]. It concerns how a place is perceived to fit an individual’s personality and desired lifestyle [80]. PD is developed through older people’s positive evaluation of how a place supports their independence through sustaining social relationships and providing accessible resources [81,82]. 

POS promotes older people’s place identity and dependence, which further influences their well-being [83,84]. Specifically, PD strengthens a sense of autonomy while place identity has positive bearings on life satisfaction and evaluation [21]. For example, everyday activities and social interactions in POS create a sense of “knowing place” and “knowing others” [85]. Familiarity with the neighborhood nurtures place identity, which further enhances the sense of environmental mastery [86]. The activities and practices that different people bring to POS provide them with shared experiences and values, crystalized as symbolic identity and emotional attachment [87]. The pleasantness of using amenities in POS contributes to PD [88], especially for older people with low SES living in crowded settlements [89]. 

Drawing on the literature, NSC and place attachment, two psychological experiences, could be possible mechanisms that explain the observed relationship between POS and older people’s well-being. Moreover, people who receive emotional and instrumental support from their neighbors may feel more attached to where they live [90]. Likewise, people who trust each other are more likely to share their daily living and place-based experiences, thus influencing their PD. Therefore, there are good reasons to believe that older people develop NSC that precedes place attachment [23,91].

## 3. Materials and Methods

### 3.1. Data Source

Data for this study were collected through a face-to-face survey in Hong Kong. A total of 501 questionnaires were collected between March 2020 and May 2021. Area-based quota sampling was used to recruit participants who lived in street block clusters (i.e., the tertiary planning units linked with census data) with a higher percentage of the older population, higher residential density, and higher or lower median monthly household income (64.67% from high and 35.33% from low income) [92]. The sampling method aims to produce sufficient representativeness of community-dwelling older people who come from typical neighborhoods that have a high percentage of aging population and high residential density [28,93,94,95]. The reason for selecting neighborhoods with both high and low income is that existing literature shows that income variation has a direct bearing on health outcomes [96]. Participants were approached through the elderly centers located in the study area [93]. To allow for a greater mix of respondents, research staff visited open spaces and parks near the elderly centers/major housing estates in the selected study areas and recruited participants. The participants of the survey were selected according to the following criteria: (1) be aged 65 or over, (2) be Cantonese (local language) speaking and able to communicate verbally, and (3) be living in one of the selected neighborhoods for at least six months. The participants’ sociodemographic characteristics are listed in Table 1. The study protocol was approved by the Human Subjects Ethics Sub-committee of the Hong Kong Polytechnic University.

### 3.2. Variables

POS is used as an independent variable. Five items were derived from the literature and findings of the pilot study [76]. Each item is scored from ‘strongly disagree’ (1) to ‘strongly agree’ (6), with a higher mean score indicating a more positive perception of POS.

Neighborhood social cohesion (NSC) and place attachment are mediators in the analytical model. NSC includes four items, which were developed based on the literature review and findings of the pilot study [76,97,98]. Each item is scored from ‘strongly disagree’ (1) to ‘strongly agree’ (6), with a higher composite score indicating a higher level of NSC.

Place Attachment includes eight items, four for place identity and four for PD [99]. Place identity and PD are measured separately despite the lack of previous attempts, as theoretically each dimension has different ways of impacting well-being. Each item in this construct is scored from ‘strongly disagree’ (1) to ‘strongly agree’ (5), with a higher mean score indicating a higher level of place identity and PD, respectively.

The well-being scale includes eleven items, including emotional (3 items), social (4 items) and psychological (4 items) well-being [100]. Each item is scored ‘never’ (0), ‘once or twice’ (1), ‘about once a week’ (2), ‘about 2 or 3 times a week’ (3), ‘almost every day’ (4), and ‘every day’ (5), with a higher mean score indicating a greater sense of well-being. An overview of each construct and its items are shown in Table 2.

Sociodemographic variables include age, gender, marital status, employment status, education attainment, housing type (i.e., public or private), living arrangement (alone or with others), and years of residence.

### 3.3. Data Analysis

**Figure 2:**
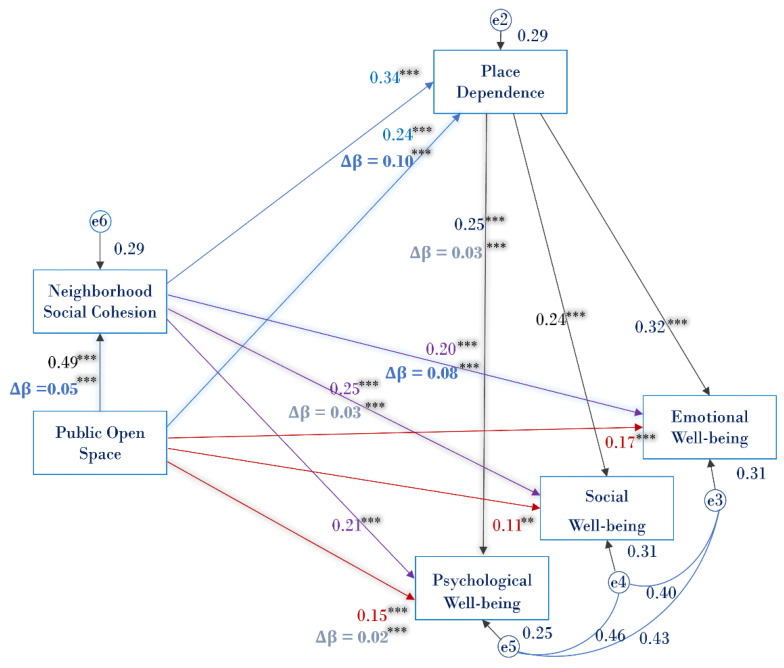
Structural equation model. ** *p* ≤ 0.01, *** *p* ≤ 0.001. Pathways highlighted in blue are stronger for the old-old group, and pathways highlighted in gray are stronger for the young-old group.

Confirmatory factor analysis (CFA) was performed to examine the reliability and validity of each construct, and adequate construct reliability and validity were presented in Table 3. The first research question was tested using SEM by Amos (version 26, IBM, New York, NY, USA), with all the variance and covariance freely estimated. Two analytical models (model fit in Table 4) were specified. Within the trial model, NSC, Place Identity, and PD were included as potential mediators. However, since the pathways linking place identity and well-being were statistically not significant in the trial model (Emotional: β = 0.026, *p* = 0.62; Social: β = 0.066, *p* = 0.21; Psychological: β = 0.087, *p* = 0.106), only NSC and PD were added in the final model (Figure 2). To estimate the direct, indirect effect and their significant level in the final model, a 95% percentile bootstrap confidence interval with 2000 iterations was specified in mediation analysis. The confidence interval excluding 0 and its *p*-value less than 0.05 indicates a significant indirect effect. To control for the influences of the demographic characteristics, all demographic characteristics are added as covariates to the dependent variables.

To address the second research question, a multigroup analysis was performed to examine the differences in the strength of associations from POS to well-being between two age groups, namely, the young-old group (aged 65 to 75, *n* = 166) and the old-old group (aged 76 to 95, *n* = 166). There were originally 335 records in the young-old group. According to Matthews (2017), 166 records were randomly selected to be included in the multigroup analysis to make the sizes of the two groups comparable [101]. The categorization of young- and old-old groups was based on the study of Neugarten (1974) [64]. The chi-square difference between the constrained and freely estimated model (with structural equivalence) indicates that the model differs across the two age groups. The path differences across the two age groups were identified using the multigroup test proposed by Gaskin & Lim (2018), comparing one pair of corresponding paths at a time [102].

## 4. Results

### 4.1. Results of SEM and Mediation Analysis

All direct and indirect effects in the specified model were significant (Table 5). In terms of the direct effects, POS has a significant and positive direct effect on three aspects of well-being (Emotional: direct effect = 0.170, CI = 0.074–0.268, *p* = 0.001; Social: direct effect = 0.114, CI = 0.022–0.204, *p* = 0.009; Psychological: direct effect = 0.152, CI = 0.050–0.244, *p* = 0.001). NSC has a significant and positive direct effect on three aspects of well-being (Emotional: direct effect = 0.203, CI = 0.105–0.303, *p* = 0.001; Social: direct effect = 0.254, CI = 0.167–0.343, *p* = 0.001; Psychological: direct effect = 0.215, CI = 0.111–0.313, *p* = 0.001). POS has significant and positive paths to NSC (direct effect = 0.492, CI = 0.412–0.558, *p* = 0.001). POS (direct effect = 0.235, CI = 0.129–0.329, *p* = 0.001) and NSC (direct effect = 0.340, CI = 0.247–0.432, *p* = 0.001) have significant and positive paths to PD.

In terms of indirect effects, significant partial mediation effects exist from POS to all aspects of well-being, through NSC and PD (Emotional: indirect effect = 0.062, CI = 0.042–0.088, p = 0.001; Social: indirect effect = 0.044, CI = 0.028–0.065, *p* = 0.001; Psychological: indirect effect = 0.037, CI = 0.023–0.056, *p* = 0.001). In short, we found that POS promotes three facets of well-being through developing NSC and, subsequently, PD, addressing the first research question.

### 4.2. Results of Multigroup Analysis

The results of the multigroup analysis (Table 6) indicate that the associations between POS and NSC (Δβ = 0.046, *p* ≤ 0.001), between POS and PD (Δβ = 0.104, *p* ≤ 0.001), as well as between NSC and emotional well-being (Δβ = 0.083, *p* ≤ 0.001) were stronger in the old-old group than for the young-old group. This means that the old-old group are more likely to acquire emotional well-being from POS through NSC.

In contrast, effects of POS (Δβ = −0.017, *p* ≤ 0.001) and PD (Δβ = −0.025, *p* ≤ 0.001) on psychological well-being and effects of NSC on social well-being (Δβ = −0.034, *p* ≤ 0.001) were stronger among the young-old group, compared with the old-old group. It indicates that POS plays a more robust role for the young-old group in achieving psychological well-being. Moreover, NSC is more relevant for the development of social well-being, for young-old people; strong NSC is likely to generate collective behaviors and social participation, which may trigger positive attitudes toward the society at large.

## 5. Discussion

This research examined the relationship between POS and older people’s well-being, considering NSC and PD as two sequential buffers. In addition, it examined the differences in the pathways connecting POS and well-being, for the old-old group and the young-old group. There are four important findings.

First, POS is positively associated with three aspects of well-being. Its association with emotional well-being is the strongest, followed by psychological and social well-being. One possible reason is that the experience in POS, particularly when older people are connected to nature, is likely to bring happiness and an instant emotional boost, leading to emotional well-being [44]. Besides, when older people find that POS is inviting and inclusive, and includes features such as friendly behaviors of others, familiar landscapes, and diverse amenities, they are more likely to feel comfortable and confident, thereby enhancing their self-esteem, satisfaction, and psychological well-being [57,58]. The inviting and inclusive POS can foster older people’s perception of social acceptance and a sense of belonging, conducive to social well-being [53,103].

Second, NSC and PD are serial mediators underlying the relationships between POS and well-being. There are two-stage psychological experiences at play when older people acquire multifaceted well-being from POS. First, NSC is developed when older people use POS. This further leads to PD and finally well-being. One possible reason might be that when older people use POS, they have more opportunities for communication and social mingling, which give rise to social connection and sense of familiarity, in line with the conclusion of Wan et al. (2021) [104]. Familiarity improves the lived experience of older people, by providing more place-based knowledge for everyday life. For example, familiarity facilitates the accumulation and exchange of community-specific information among residents, conducive to PD as residents know places to eat and visit, places that sell fresh or cheaper goods, and places for events and recreation [75,105,106]. This finding highlights that provision of POS creates a place that is not only relationship-rich, but also functionally important to daily living.

Third, the findings show that place identity has no mediation effect in the POS–well-being relationship as the path from place identity to well-being is insignificant. This finding seems unique in Hong Kong and appears to contrast with prior research [107], which suggested that access to blue and green features in POS promotes place identity, thereby contributing to well-being. One possible reason might be that older people evaluated place attachment by putting a lot of weight on PD. The follow-up interviews provided supplementary proof. When they were asked if they would like to move to a different place of residence if they had a choice, most people preferred their current residence. There was a sign of place attachment, but most of the reasons for place attachment were related to PD. Older people are dependent on their current residence because it fulfills their daily life needs, such as being close to cafeterias, community centers, shops, parks, and public transport. The variety of and accessibility to community facilities creates functional, more than emotional, attachment to where they live [108].

Another possible reason is that from place identity to well-being, social participation in the community may be an underlying mechanism [109,110]. Place identity is an inspiration that triggers actions to participate in community affairs. This is because when people identify themselves strongly with where they live, they are motivated to seek, stay in, protect and improve the places that are meaningful to them, and their involvement and contribution will be rewarded with well-being [110]. According to He et al. (2018) and Chou (2018), many older people in Hong Kong do not have enough opportunities for community participation due to financial constraints and insufficient accessibility of safe transportation [111,112,113,114]. Moreover, the research was conducted during the COVID-19 pandemic. Apart from regular meetings with older people, elderly centers suspended most activities. This may have influenced the relationship between place identity and well-being.

Lastly, the results of the multigroup analysis suggest that the strength of association between POS and well-being varies in different stages of aging (i.e., young-old and old-old). The pathway from POS to emotional well-being via NSC is stronger for the old-old group. This makes sense. First, as mentioned in the literature [61,65], the old-old group is better at emotion regulation. They tend to acquire better emotional well-being from POS. Second, as people age, their activity space and social networks are relatively stable and fixed to those they are familiar with [115]. Their increased familiarity with old places and old folks tends to provide them more NSC, for getting trust and mutual help [82,106,116,117]. Accordingly, POS has a greater influence on PD for the old-old group.

POS is more important for the young-old group to develop their psychological well-being, as the direct effect of POS on psychological well-being is stronger. One possible reason might be that the young-old group has fewer physical and cognitive constraints, thereby participating in various activities and traveling a longer distance. As such, they have more intense environmental encounters and use POS outside their neighborhood more often. They spend more time outdoors, conduct diverse physical activities in various localities, and develop different ways of natural contact [59,60,118,119]. These multiple encounters provide more psychological benefits to the young-old group.

This study adds value to the social determinants of health theory. It identifies how POS, an important environmental attribute in neighborhoods, influences older people’s psychological experiences, which ultimately promotes well-being. The study calls for more attention to the psychological benefits underlying the links between environment and well-being. This helps better understand the social determinants of health theory in terms of how older people receive and translate the socio-spatial features of the environments into positive outcomes. However, the empirical results reported herein should be interpreted in light of some limitations. First, the present study only utilized subjective measures of POS. Although the perceptions of environments are relevant to psychological experiences, it is important to include the objective features of environments into the analysis model as it is interesting to compare if people living in the same environmental settings develop varied psychological experiences and well-being outcomes. For example, open data can be used to derive objective measurements of POS (e.g., objective accessibility); wearable sensors and eye-tracking systems can be used to assess how POS impacts individuals’ attention and mobility behaviors. Second, analysis results and conclusions were drawn from a cross-sectional study, which may not provide a complete picture of their causal relationships, particularly considering that the development of NSC and PD needs time. Future studies are recommended to use a longitudinal methodology to explore the casual relationships between the POS and well-being and test the effects of psychological experiences underlying the observed relationships.

## 6. Conclusions

This paper extends the focal points of well-being research through a cross-sectional study of an ultra-dense metropolis, Hong Kong. The findings indicate that POS can support the well-being of older people through developing two-stage psychological experiences that include NSC and PD. Results of the multi-group analysis suggest that there are differences between young-old and old-old residents in terms of how they acquire multifaceted well-being from POS. For old-old, using POS maintains emotional well-being; for young-old group, POS is more likely to generate psychological well-being.

This study has significant implications for urban planners, policymakers, and community workers trying to support older people when aging in place. The results provide empirical evidence that POS is more than a collection of physical attributes but an important community asset for generating social and psychological values. It contributes to older people’s social cohesion and the bonding ties to where they live. The accessibility of parks, safe and sufficient pedestrian spaces, green features, and attractive natural scenery are important public assets and resources for promoting well-being. POS is essential in terms of its aesthetic values and social functions to bring people together with quality social relations and cohesion. Hence, providing POS in densely populated urban environments can produce places that are relationship rich [14]. In addition, the planning and design of POS should emphasize the variety of facilities and amenities, as they not only support daily life but also contribute to the development of PD, upon which older people acquire autonomy and environmental mastery.

## Figures and Tables

**Table 1:** Participants’ Sociodemographic Characteristics.

Characteristics	Young-Old (*n* = 335)	Old-Old (*n* = 166)
**Gender**		
Female	239 (71.3%)	103 (62.0%)
Male	96 (28.7%)	63 (38.0%)
**Age (Years)**	Mean: 69, SD: 3.1	Mean: 83, SD: 4.6
**Marital status**		
Divorced/separated	28 (8.4%)	7 (4.2%)
Married	214 (63.9%)	76 (45.8%)
Never Married	40 (11.9%)	5 (3%)
Widowed	53 (15.8%)	78 (47.0%)
**Educational attainment**		
High school	7 (2.1%)	3 (1.8%)
None/preschool	21 (6.3%)	51 (30.7%)
Primary school	84 (25.1%)	58 (34.9%)
Secondary school	151 (45.1%)	43 (25.9%)
Tertiary education or above	72 (21.5%)	11 (6.6%)
**Housing type**		
Private	183 (54.6%)	70 (42.2%)
Public	152 (45.4%)	96 (57.8%)
**Years of residence**	Mean: 27, SD: 13.6	Mean: 37, SD: 17.6
**Living arrangement**		
Living alone	80 (23.9%)	59 (35.5%)

**Table 2:** Constructs and Items.

Items	Cronbach’s Alpha	Mean Score
**Public Open Space (POS)**	0.862	4.40
The natural scenery or scenic spots in the area attract me very much.		4.30
I like the parks near the estate or gardens on the street.		4.43
There are plenty of public open spaces for walking in the community.		4.45
The neighborhood is tree-lined with many plants.		4.36
The community has plenty of public space, and I often go downstairs for a walk or exercise.		4.48
**Neighborhood Social Cohesion (NSC)**	0.839	4.19
Neighbors get along well with each other.		4.48
Neighbors are willing to help each other.		4.17
I will talk to the neighbors I meet when I walk around the area.		4.19
If something important happens in this community, I will know.		3.91
**Place Identity (PI)**	0.826	3.84
The place where I live is of great significance to me.		4.10
I am very dependent on the place where I live.		3.77
I have a great sense of identity with the place where I live.		3.74
I have a special connection with the place where I live and people here.		3.73
**Place Dependence (PD)**	0.887	4.10
I like where I live more than other places.		4.17
The place where I live is more satisfactory to me than other places.		4.12
Living here is more important than living elsewhere.		4.01
I will not select another place to replace where I live.		4.09
**Emotional Well-being (W1)**	0.895	3.65
Happy and joyful.		3.57
Life is fun.		3.58
Satisfactory.		3.81
**Social Well-being (W2)**	0.798	3.28
I belong to a certain society (for example, a group of people or a sports group).		3.33
Our society is developing in a better direction for everyone.		3.15
People are basically good.		3.54
The operation of society is effective.		3.10
**Psychological Well-being (W3)**	0.808	3.87
I love most about my characters.		3.86
I feel warm and confident when I meet people and they return this warmth to me.		3.79
My experience makes me a better person.		4.06
I dare to think and express my own ideas.		3.75

**Table 3:** Results of construct reliability and validity.

Range of Standardized Path Loadings		CR	AVE	1	2	3	4	5	6
Model fit of the measurement model (Normed x^2^ = 2.453; CFI = 0.949; TLI = 0.941; RMSEA = 0.054; SRMR = 0.051)									
1. NSC	0.596–0.871	0.85	0.59	0.768					
2. POS	0.687–0.803	0.864	0.561	0.537 ***	0.749				
3. W1	0.833–0.89	0.897	0.744	0.475 ***	0.443 ***	0.863			
4. W2	0.529–0.806	0.823	0.543	0.479 ***	0.395 ***	0.625 ***	0.737		
5. W3	0.661–0.767	0.812	0.52	0.461 ***	0.427 ***	0.691 ***	0.670 ***	0.721	
6. PD	0.657–0.91	0.897	0.689	0.510 ***	0.451 ***	0.505 ***	0.479 ***	0.488 ***	0.83

Note: *** *p* ≤ 0.001. Diagonal elements represent the square root of the average variance extracted (AVE), while off-diagonal elements represent the correlations. For adequate discriminant validity, diagonal elements should be greater than corresponding off-diagonal elements. To ensure construct validity and reliability, one item was removed from POS, and three items were removed from the well-being scale for further analysis.

**Table 4:** Model fit of the structural model.

Measure	Estimate	Threshold	Interpretation
CMIN	37.694	--	--
DF	26.000	--	--
CMIN/DF	1.450	Between 1 and 3	Excellent
CFI	0.992	>0.95	Excellent
SRMR	0.030	<0.08	Excellent
RMSEA	0.030	<0.06	Excellent
PClose	0.953	>0.05	Excellent

**Table 5:** Mediation analysis results.

Pathway	Indirect Effects	BC 95% CI		*p*-Value
		Lower	Upper	
POS → NSC → PD → W1	0.062	0.042	0.088	0.001
POS → NSC → PD → W2	0.044	0.028	0.065	0.001
POS → NSC → PD → W3	0.037	0.023	0.056	0.001

**Table 6:** Statistical comparison for two age groups.

Path Name	Old-Old	Young-Old	Difference in Betas
POS → NSC	0.536 ***	0.489 ***	0.046 ***
POS → PD	0.295 ***	0.190 ***	0.104 ***
NSC → W2	0.229 **	0.263 ***	−0.034 ***
NSC → W1	0.249 **	0.166 **	0.083 ***
PD → W3	0.237 ***	0.261 ***	−0.025 ***
POS → W3	0.128 †	0.145 **	−0.017 ***

Note: † *p* ≤ 0.1, ** *p* ≤ 0.01, *** *p* ≤ 0.001. Insignificant results were removed.

## Data Availability

The data presented in this study are available on request from the corresponding author.
